# Simulation Studies on the Effect of Material Characteristics and Runners Layout Geometry on the Filling Imbalance in Geometrically Balanced Injection Molds

**DOI:** 10.3390/polym11040639

**Published:** 2019-04-08

**Authors:** Krzysztof Wilczyński, Przemysław Narowski

**Affiliations:** Faculty of Production Engineering, Polymer Processing Department, Warsaw University of Technology, 02-524 Warsaw, 85 Narbutta, Poland; przemyslaw.narowski@pw.edu.pl

**Keywords:** injection molding, filling imbalance, 3D modeling

## Abstract

Simulation studies were performed on filling imbalance in geometrically balanced injection molds. A special simulation procedure was applied to simulate properly the phenomenon, including inertia effects and 3D tetrahedron meshing as well as meshing of the nozzle. The phenomenon was investigated by simulation using several different runner systems at various thermo-rheological material parameters and process operating conditions. It has been observed that the Cross-WLF parameters, index flow, critical shear stress (relaxation time), and zero viscosity, as well as thermal diffusivity and heat transfer coefficient strongly affect the filling imbalance. The effect is substantially dependent on the runners’ layout geometry, as well as on the operating conditions, flow rate, and shear rate. The standard layout geometry and the corrected layout with circled element induce positive imbalance which means that inner cavities fills out faster, and it is opposite for the corrected layouts with one/two overturn elements which cause negative imbalance. Generally, for the standard layout geometry and the corrected layout with circled element, an effect of the zero shear rate viscosity η_0_ is positive (imbalance increases with an increase of viscosity), and an effect of the power law index *n* and the relaxation time λ is negative (imbalance decreases with an increase of index *n* and relaxation time λ). An effect of the thermal diffusivity α and heat transfer coefficient *h* is negative while an effect of the shear rate is positive. For the corrected layouts with one/two overturn elements, the results of simulations indicate opposite relationships. A novel optimization approach solving the filling imbalance problem and a novel concept of global modeling of injection molding process are also discussed.

## 1. Introduction

**Figure 1:**
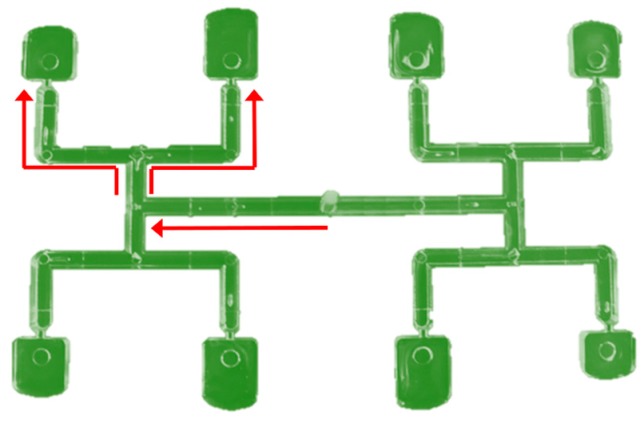
Filling imbalance in geometrically balanced injection molds.

The phenomenon of filling imbalance in multi-cavity injection molds equipped with geometrically balanced runner systems is a serious problem in the injection molding process. This phenomenon can be observed in H-type runner layouts, e.g., with eight cavities (Figure 1). Additionally, it is generally established that the filling imbalance is caused by the shear gradients in the flow through the runners that lead to non-linear and non-symmetrical distributions of temperature and viscosity. This is strongly influenced and complicated by the geometry of the runners, the thermo-rheological material characteristics, and the process parameters of injection molding [1,2,3,4,5,6].

The filling imbalance has been examined extensively by scientists and engineers, e.g., [1,2,3,4,5,6,7,8,9,10,11,12,13,14,15,16,17,18,19,20,21,22,23,24,25]. Up to now, there have been no universal and commonly accepted solutions of this problem. A detailed discussion of the literature review has been recently presented by the authors of this paper [1].

The filling imbalance has been also studied in the polymer industry. A good example of injection molding process very sensitive to filling imbalance is the manufacturing of lenses [26]. An importance of the effect of filling imbalance on the mold cost and energy consumption has been presented in [27].

The fundamental research on the problem had been carried out by Beaumont group who developed melt rotation technology to minimize the filling imbalance [2,3,4,5]. Beaumont proposed several runner layouts, however, these concepts were not subjected to systematic simulations or experimentations.

Recently, extensive experimental studies on the filling imbalance have been performed by the authors [1]. Balancing the polymer melt flow between cavities has been investigated at various operating conditions using different runner systems. The experiments indicate that the injection rate, the mold and melt temperatures substantially affect the filling imbalance. Additionally, the filling imbalance is strongly dependent on the runners’ layout geometry. It is worth noticing that filling imbalance has never been eliminated completely.

The flows of molten polymers in injection molds are unsteady, non-Newtonian and non-isothermal flows occurring at very high rates of deformation. The primary cause of the filling imbalance are nonlinear distributions of the polymer melt velocity and shear rate, as well as the melt temperature which determine the melt viscosity distribution. Such complex flows can only be modeled numerically, e.g., using FEM (finite element method) computations.

First, numerical simulations of the filling imbalance [28,29,30,31] did not allow to predict the phenomenon properly. The use of 2D or 2.5D approaches did not allow to solve the problem. Thus, new concepts were necessary which included inertia effects and 3D, non-isothermal, and non-Newtonian flow [32,33]. Several simulation tests have confirmed the effectiveness of these methods [1,34,35,36,37].

When modeling the flow of molten polymers in injection molds, the initial flow parameters, e.g., melt temperature and pressure, are basically unknown and usually assumed without much justification. Therefore, a comprehensive approach to modeling the injection molding process is proposed which includes the polymer melt flow both in the plasticating unit as well as in the mold.

**Figure 2:**
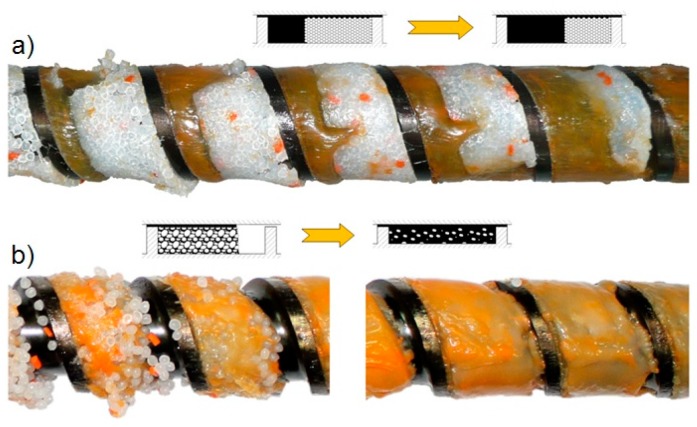
Mechanism of polymer melting in the single screw extruder on an example of extrusion of polypropylene: (**a**) flood fed extrusion, classical Tadmor mechanism, and (**b**) starve-fed extrusion, two-stage mechanism [46].

Modeling of the polymer melt flow in the plasticating unit of the injection molding machine is based on the modeling of the extrusion process. The first fundamental research in this area has been carried out by Tadmor et al. [38,39] for single screw extrusion. These studies have been later extended to twin screw extrusion, both co-rotating [40,41] and counter-rotating [42,43,44], and starve-fed single screw extrusion [45,46], and finally have been successfully used for modeling of the injection molding process [47,48,49,50]. The modeling research on the screw processing has been summarized by Altinkaynak et al. [51] and Wilczyński et al. [52]. The mechanism of polymer melting in the single screw extruder has been depicted in Figure 2, both for flood fed extrusion (classical Tadmor mechanism) and for starve fed extrusion (two-stage mechanism).

**Figure 3:**
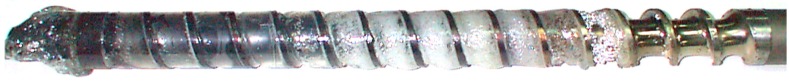
Mechanism of polymer melting in the injection molding machine on an example of injection molding of polystyrene (unpublished).

The authors have carried out an experimental (unpublished) research on the melting mechanism of polymers in the injection molding machine (Figure 3). It is interesting to note that the melting in the injection molding machine occurs to some extent according to the Tadmor mechanism, however, with clearly visible starvation.

The existing models of injection molding process (plasticating unit) [47,48,49,50] differ from the extrusion models in that they involve the static and dynamic phases of melting (stationary and rotating screw) with an axial screw movement. However, they assume the screw channel is fully filled with a material as in the flood fed extrusion (Figure 2a). According to our observation (Figure 3) it is not true, and starvation is here clearly seen as in the starve fed extrusion (Figure 2b). Thus, it may be supposed that the solid conveying section and melting section should be modelled similarly to the starve-fed extrusion [45,46], of course accepting the other assumptions valid for the injection molding process.

Recently, novel concepts of modeling of polymer melting have been presented [51,53]. These involve the modeling of a two-phase flow (solid/melt) as a single-phase flow, and the resulting temperature distribution determines the boundary between melt and solid. This concept is independent on the screw geometry, and does not require any knowledge of the melting mechanism. More advanced concepts include DEM modeling (discrete element method) for solid conveying and FEM modeling (finite element method) for melting and melt flow. It is also worth highlighting that the full 3D simulations are well developed for die extrusion flows including viscoelastic models [54].

So far, the filling imbalance has been mainly studied experimentally and in a much less extent by simulation (for the standard systems and for the basic melt rotation systems, only). In this paper, an extensive simulation study on the filling imbalance has been performed for several different runner systems, at various thermo-rheological material parameters and process operating parameters, to explain the phenomenon. The Autodesk Simulation Moldflow Insight 2014 software has been used for simulations [26]. According to our knowledge it is the most comprehensive simulation and modeling research on the filling imbalance with particular emphasis on material and geometrical effects.

The novel approaches solving the imbalance problem have been also presented including an optimization concept with the use of advanced process modeling, as well as the concept of global modeling of the injection molding process.

## 2. Process Simulation

### 2.1. Simulation Program

Process simulations have been carried out to study an effect of the material characteristics and runners’ layout geometry on the filling imbalance in geometrically balanced injection molds.

**Figure 4:**
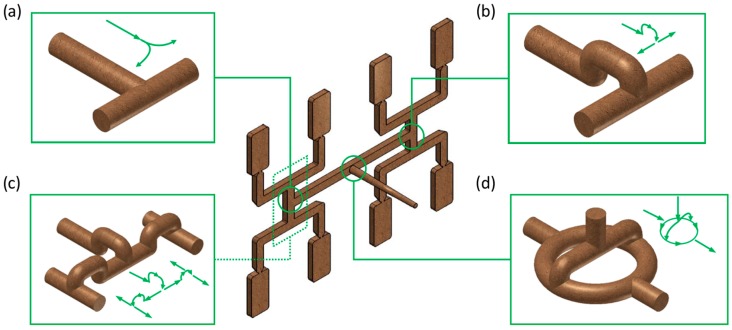
Geometry of runner layouts: (**a**) standard *GS*, (**b**) one correction element *G1*, (**c**) two correction elements *G2*, and (**d**) circled element *G3*.

Simulations have been performed for an eight-cavity injection mold of “H-type” runners’ layout equipped with inserts of different geometry. A standard geometry *GS* has been used, as well as three overturn geometries: *G1* with one correction element, *G2* with two correction elements, and *G3* with circled element, These are depicted in Figure 4.

An effect of the thermo-rheological material characteristics has been studied under different operating conditions, at various injection rates (flow rates). The processing parameters are presented in Table 1.

### 2.2. Material

We have used polybutylene terephthalate PBT Valox 337 in the study (manufactured by SABIC, Riyadh, Saudi Arabia) as the reference polymer to compare the research results with recently performed experimentations [1] and other simulations [31,32]. The material characteristics has been collected in Table 1 [28].

The viscosity of the material was modeled using the non-Newtonian Cross-WLF model which describes the shear thinning behaviour and the temeperature effect, and it may be presented in the following form:

(1) $$\eta =\frac{\eta_{0}}{1+{\left(\frac{\eta_{0}\dot{\gamma }}{\tau^{*}}\right)}^{1-n}}$$

(2) $$\eta_{0}=D_{1}\exp\left[-\frac{A_{1}\left(T-T^{*}\right)}{A_{2}+\left(T-T^{*}\right)}\right]$$

where η is the melt viscosity, Pa∙s; $\eta_{0}$ is the zero viscosity, Pa∙s; $\dot{\gamma }$ is the shear rate, s^−1^; $\tau^{*}$ is the critical shear stress, Pa; $\eta_{0}$/$\tau^{*}$ is the relaxation time λ, s; *n* is the power law index, dimensionless; *T* is the temperature, K; and *D_1_*, *A_1_*, *A_2_*, and T^*^ are the parameters of the Cross-WLF equation.

**Figure 5:**
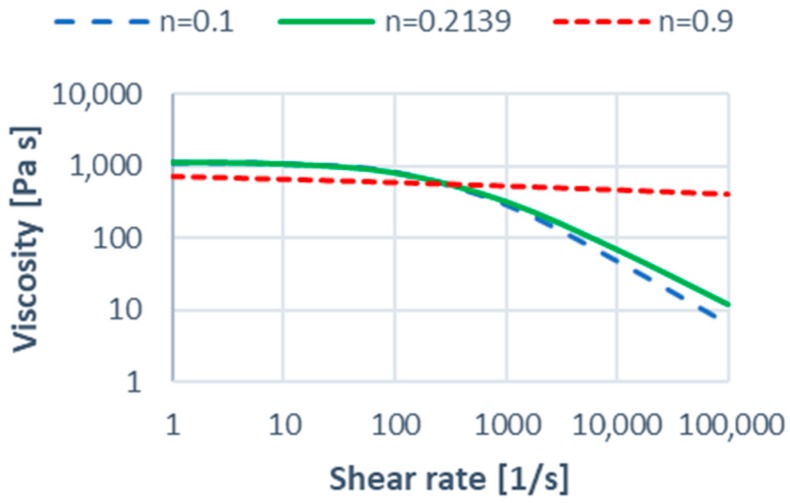
Effect of the power-law index *n* on the viscosity curve in relation to PBT Valox 337 (*n* = 0.2139).

**Figure 6:**
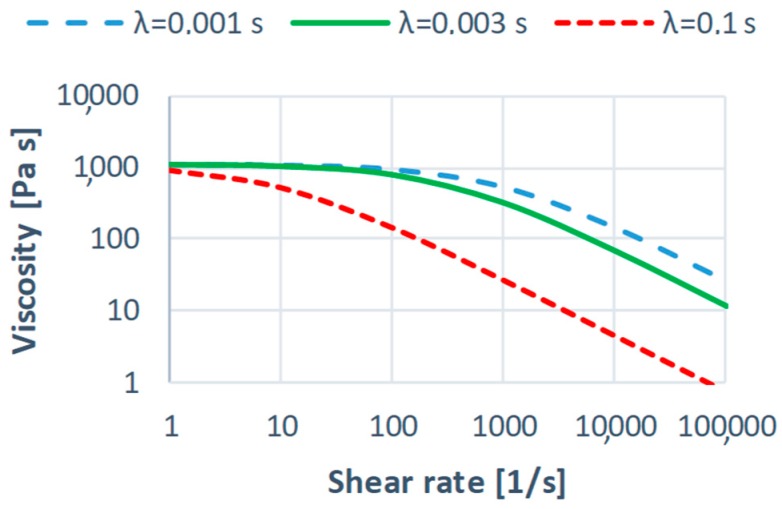
Effect of the relaxation time λ = $\eta_{0}$/$\tau^{*}$ on the viscosity curve in relation to PBT Valox 337 (λ = 0.003 s, calculated for *T* = 252 °C).

**Figure 7:**
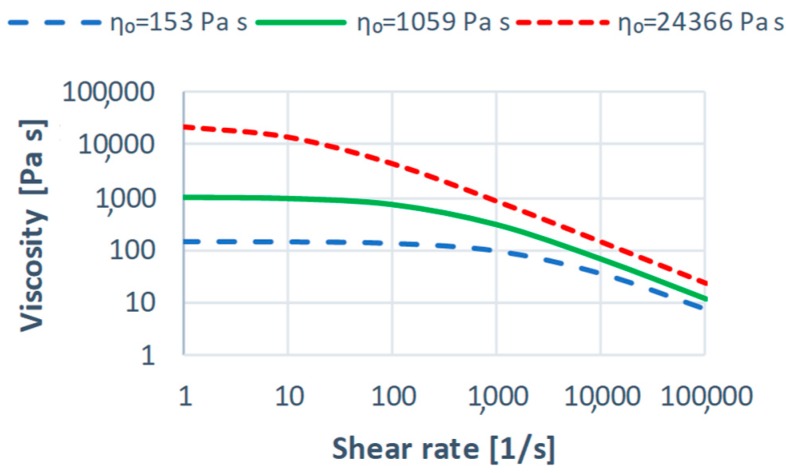
Effect of the zero shear viscosity $\eta_{0}$ on the viscosity curve in relation to PBT Valox 337 ($\eta_{0}$ = 1059 Pa·s, calculated for *T* = 252 °C).

An effect of the Cross-WLF parameters on the shear viscosity curves is depicted in Figure 5, Figure 6 and Figure 7 in relation to the parameters of the reference material (Table 1).

The two limiting values of the Cross-WLF model parameters have been selected as the limiting values of these parameters of the materials available in the Autodesk Moldflow database. The different melt temperature was set to change the zero shear viscosity, and the relaxation time λ was set by changing τ*.

A sharp drop of the shear viscosity η for low values of the power law index *n* (shear thinning) at higher shear rates $\dot{\gamma }$ (typical for injection molding) is clearly visible (Figure 5). An increase in the relaxation time λ also leads to the significant lowering of the viscosity η at higher shear rates $\dot{\gamma }$. An effect of the zero shear viscosity $\eta_{0}$ on the viscosity curve is greater for lower shear rates $\dot{\gamma }$, however, also significant in the injection molding shear rate range. In summary, it can be stated that rheological parameters of the material have a significant impact on the shear viscosity η in the typical for injection molding range of the shear rate, that is $\dot{\gamma }$ = (10^3^–10^4^, 10^5^) s^−1^. This affects the filling imbalance phenomenon.

### 2.3. Procedure of Estimation of Filling Imbalance

**Figure 8:**
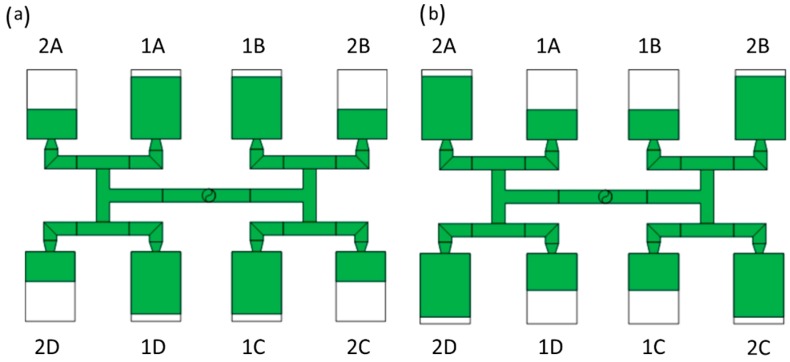
The concept of mass filling imbalance coefficient: (**a**) positive imbalance, *I_m_* *> 0 (m_1_ > m_2_)*, faster filling of inner cavities; and (**b**) negative imbalance, *I_m_* *< 0 (m_1_ < m_2_)*, faster filling of outer cavities.

The mass filling imbalance coefficient *I_m_* [2] has been used to estimate the filling imbalance which is defined by:

(3) $$I_{m}=\left(1-\frac{m_{2}}{m_{1}}\right)\times 100\%$$

where *I_m_* is the mass filling imbalance coefficient, *m_1_* is the mass of the polymer in the inner cavities, and *m_2_* is the mass of the polymer in the outer cavities (Figure 8).

The mass filling imbalance coefficient is positive when the inner cavities (1) are filled out faster *(I_m_ > 0)*, and it is negative when the outer cavities (2) are filled out faster *(I_m_ < 0)*.

### 2.4. Simulation Technique

**Figure 9:**
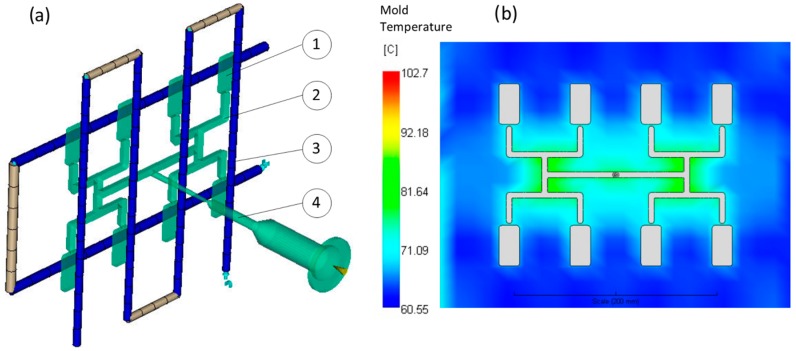
Simulation procedure: (**a**) simulation model: 1—cavity, 2—runners, 3—cooling channels, 4—nozzle, and (**b**) mold temperature distribution.

Simulations have been performed using the Moldflow (San Rafael, CA, USA) Insight 2014 program [28]. A special simulation technique has been applied to study the filling imbalance phenomenon. It embraces the geometrical model design of the flow space, meshing technique and definition of the solver parameters:

**_–_** the geometrical model of the flow space should cover all the flow and the flow environment components, i.e., the cavities, the runners, the moldbase, and the cooling channels, as well as the nozzle where the filling imbalance starts to develop (Figure 9a), and the proper design of cooling channels ensures an even temperature distribution of the mold (Figure 9b).

**_–_** the meshing should be performed using the 3D tetrahedron mesh elements to properly model the asymmetric distributions of the flow parameters, e.g., shear rate and temperature, and the maksimum mesh size should not exceed the *f* value (*f = D/2N, D*–characteristic dimension of the channel, cavity or runners, *N*–number of layers), and the number of layers across the part thickness should not be lower than 12 (the only fine solid mesh is able to catch the shear rate distribution properly across the runner), 

**_–_** when modeling and using the equation of motion, the inertia components should be taken into account which improves the prediction of the polymer melt front.

It is important to know, that the modeling of filling imbalance is time consuming and usually takes several hours (6–8) for about 1,500,000 tetrahedrons. The simulations were performed using an HP Z640 workstation; Intel^®^ Xeon^®^ E5-2667 v4 (3.2 GHz, 25 MB cache, 8 cores, Intel^®^ vPro™) equipped with 128 GB DDR4-2400 ECC registered RAM.

**Figure 10:**
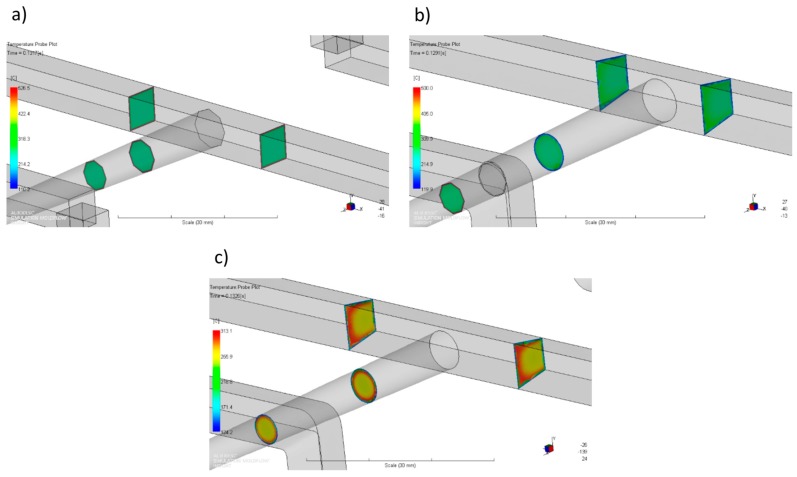
Meshing for runners modeling: (**a**) 1D conventional beam elements, (**b**) combination of 1D/3D elements, and (**c**) 3D meshing.

It is also important to know that the conventional 1D beam elements are not suitable for the runner modeling (Figure 10a). These cannot catch the non-symmetrical temperature distribution in the runner cross section. Combination of the conventional 1D beam elements with 3D elements is also not recommended. At the transition cross-section between these two meshes the shear rate parameters are not transferred properly (Figure 10b). Only the 3D mesh is able to simulate the shear induced filling imbalance properly (Figure 10c).

## 3. Results

Simulations have been performed for all the runner layouts (Figure 4), i.e., for a standard geometry and for three overturn geometries, at various injection rates *V_inj_*, for different rheological and thermal parameters of the material. An effect of the parameters of the Cross-WLF model, i.e., the zero shear rate viscosity η_0_, the power law index *n* and the relaxation time λ, has been studied as well as an effect of the thermal properties of the material, i.e., the thermal diffusivity α and the heat transfer coefficient *h* has been simulated. Obviously, while considering the effect of the selected parameter, the other were kept constant.

Mass filling imbalance coefficient *I_m_* has been plotted against injection rate *V_inj_*, and distributions of the polymer melt velocity have been depicted for all the simulation cases.

The surface areas of the filled parts of cavities have been measured at the stage of 80% of mold filling, and these areas have been inserted in places of m_1_ and m_2_ (Equation (3)). It has been assumed the density is constant.

First, an effect of the rheological parameters has been studied, then an effect of the thermal parameters has been discussed and, finally, an effect of the runner geometry for selected rheological and thermal parameters has been considered.

### 3.1. Effect of Rheological Parameters

**Figure 11:**
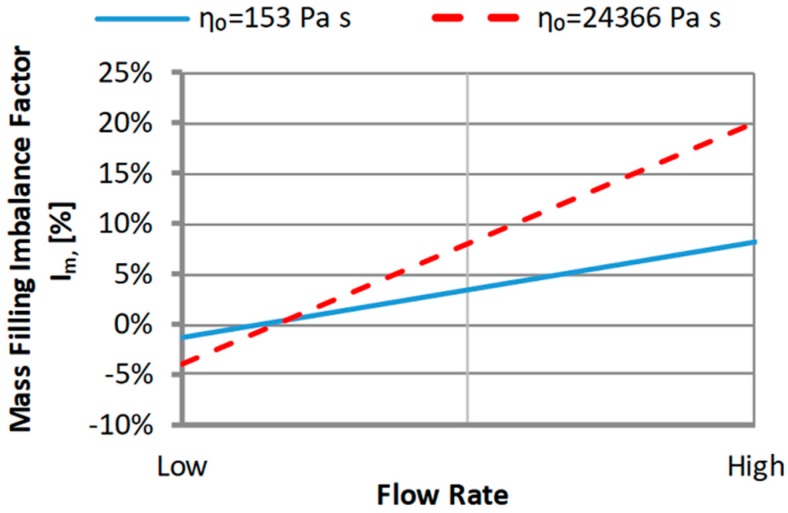
An effect of the zero shear viscosity η_0_ on the mass filling imbalance factor *I_m_* at various injection rates (Low = 5 cm^3^/s, High = 500 cm^3^/s).

**Figure 12:**
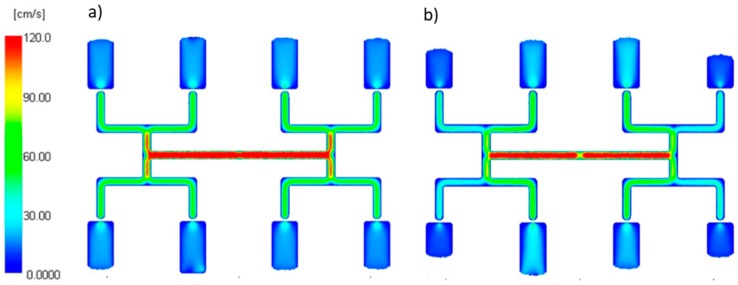
Velocity distribution for different zero shear viscosity at high injection rate: (**a**) η_0_ = 153 Pa·s, (**b**) η_0_ = 24,366 Pa·s.

**Figure 13:**
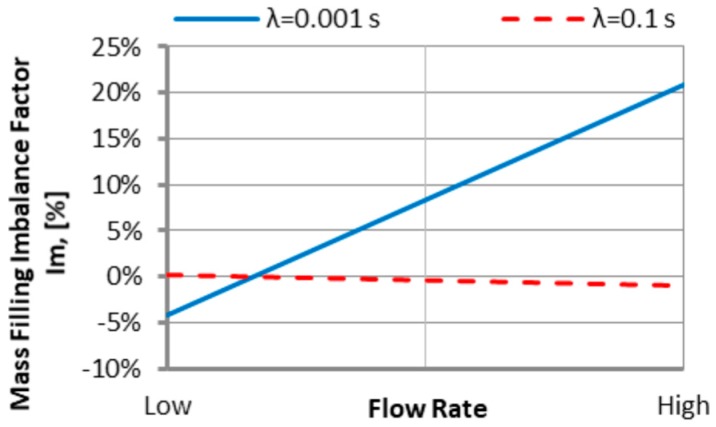
An effect of the relaxation time λ on the mass filling imbalance factor *I_m_* at various injection rates (Low = 5 cm^3^/s, High = 500 cm^3^/s).

**Figure 14:**
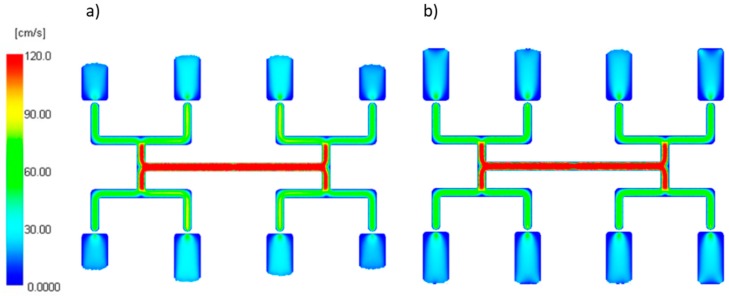
Velocity distribution for different relaxation time at high injection rate: (**a**) λ = 0.001 s, (**b**) λ= 0.1 s.

**Figure 15:**
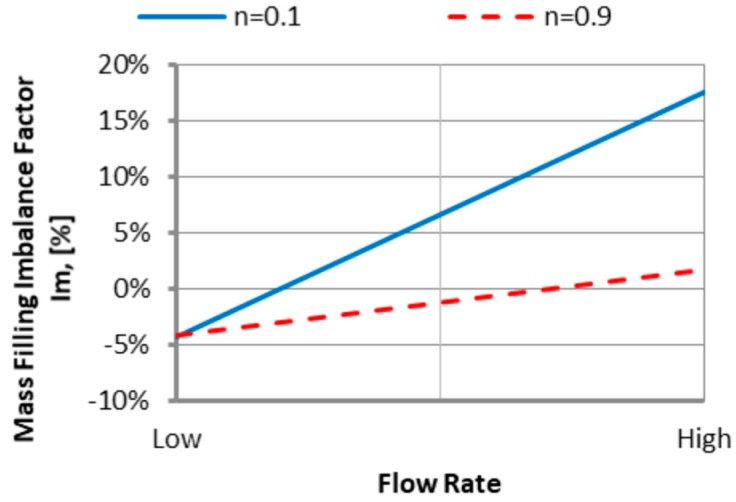
An effect of the power law index *n* on the mass filling imbalance factor *I_m_* at various injection rates (Low = 5 cm^3^/s, High = 500 cm^3^/s).

**Figure 16:**
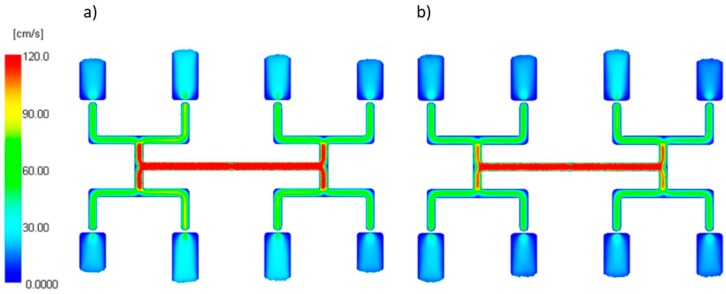
Velocity distribution for different power law index at high injection rate: (**a**) *n* = 0.1, and (**b**) *n* = 0.9.

An effect of the rheological parameters of the material on the filling imbalance has been depicted in Figure 11, Figure 12, Figure 13, Figure 14, Figure 15 and Figure 16. Here, as an example, the simulation results for the standard geometry of the runners’ layout have been presented for the lowest and the highest values of the parameters studied (Figure 5, Figure 6 and Figure 7).

#### 3.1.1. The Zero Shear Viscosity

An effect of the zero shear rate viscosity η_0_ on the filling imbalance for the standard geometry of the runners layout is generally positive which means that the inner cavities fills out faster, and this significantly increases with an increase of the injection rate (Figure 11). The imbalance increases when the viscosity increases. Higher viscosity means the higher energy dissipation, so it means the flow with higher temperature gradients which promotes the filling imbalance. Velocity distributions and the flow front advancement (Figure 12) clearly confirm this observation, and the higher velocity gradients are observed when the viscosity increases.

#### 3.1.2. The Relaxation Time

An effect of the relaxation time λ on the filling imbalance is positive for low λ values, and then the inner cavities fills out faster, and this increases significantly with an increase of the injection rate (Figure 13). However, the imbalance decreases almost to zero when the relaxation time increases. This results from a substantial viscosity decrease (Figure 6), i.e., the lower energy dissipation, and the flow with lower temperature gradients which does not promote the filling imbalance. Velocity distributions and the flow front advancement (Figure 14) confirm this observation, and the higher velocity gradients are observed for the lower relaxation time.

#### 3.1.3. The Power Law Index

An effect of the power law index *n* is also positive for low *n* values which means the inner cavities fills out faster, and this increases substantially with an increase of the injection rate (Figure 15). However, the imbalance decreases basically to zero when the power low index increases. This is a clear proof that the imbalance is greater when the fluid is more non-Newtonian. Velocity distributions and the flow front advancement (Figure 16) confirm this observation, and the higher velocity gradients are observed for the lower power law index.

### 3.2. Effect of Thermal Parameters

**Figure 17:**
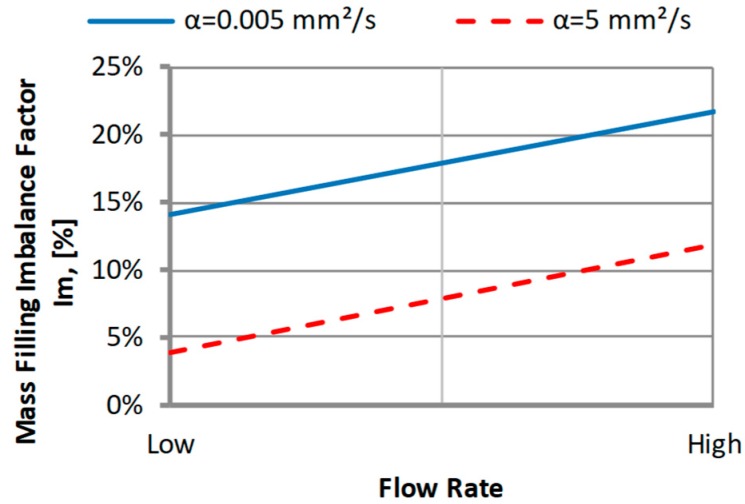
An effect of the thermal diffusivity α on the mass filling imbalance factor *I_m_* at various injection rates (Low = 5 cm^3^/s, High = 500 cm^3^/s).

**Figure 18:**
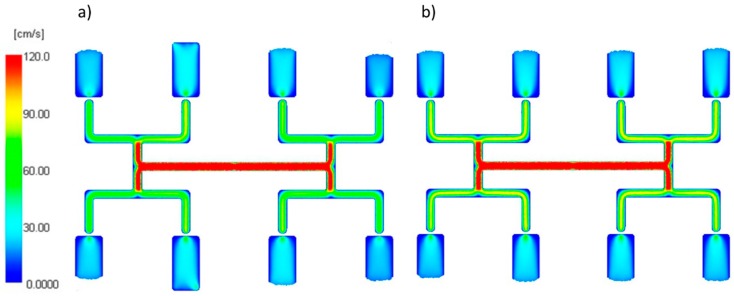
Velocity distribution for different thermal diffusivity at high injection rate: (**a**) *α* = 0.005 mm^2^/s, and (**b**) *α* = 5 mm^2^/s.

**Figure 19:**
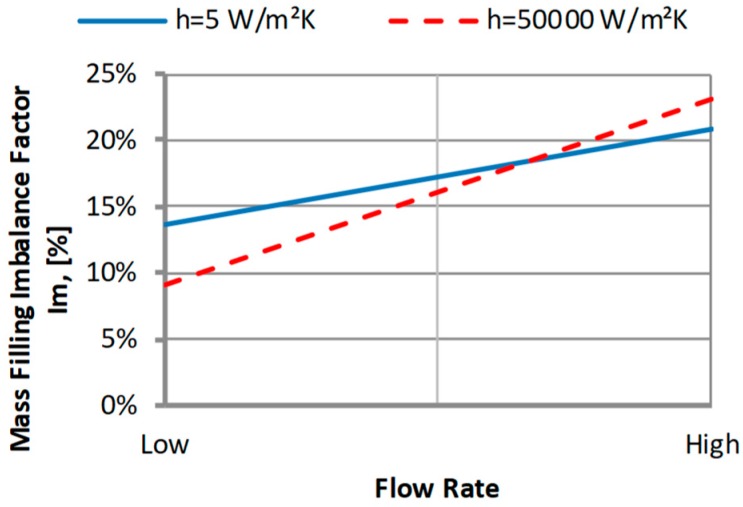
An effect of the heat transfer coefficient *h* on the mass filling imbalance factor *I_m_* at various injection rates (Low = 5 cm^3^/s, High = 500 cm^3^/s).

**Figure 20:**
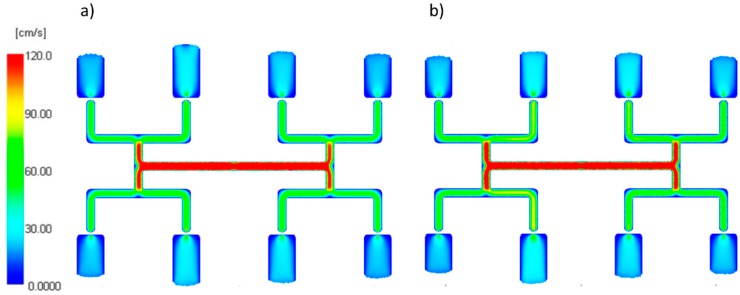
Velocity distribution for different heat transfer coefficient at high injection rate: (**a**) *h* = 5 W/(m^2^K), and (**b**) *h* = 50,000 W/(m^2^K).

An effect of the thermal parameters of the material on the filling imbalance has been depicted in Figure 17, Figure 18, Figure 19 and Figure 20. Here, as an example, the simulation results for the standard geometry of the runners’ layout have been presented.

#### 3.2.1. The Thermal Diffusivity

The thermal diffusivity *α* (the middle value *α* = 0.112 mm^2^/s) which is a combination of the thermal conductivity, density, and specific heat capacity, was set by changing the thermal conductivity.

An effect of the thermal diffusivity α on the filling imbalance is positive for low and high α values which means that inner cavities fills out faster, This positive effect significantly increases with an increase of the injection rate (Figure 17). However, the imbalance decreases when the thermal diffusivity increases. The results of the simulation show that a more intense heat exchange, i.e., higher α promotes balancing the flow. This is clearly confirmed by velocity distributions and the flow front advancement (Figure 18).

#### 3.2.2. The Heat Transfer Coefficient

The heat transfer coefficient describes the heat transfer between the mold and the polymer melt. The middle value *h* = 5000 W/(m^2^K) has been assumed by default (Autodesk Moldflow).

An effect of the heat transfer coefficient *h* on the filling imbalance is also positive which means that inner cavities fills out faster. However, the relationship between the imbalance, the heat transfer coefficient, and the flow rate is not very clear (Figure 19). For low *h* values, the imbalance is higher for the low flow rate, while for high *h* values, the imbalance is higher for the high flow rate. This is confirmed by Figure 20. Additionally, it is worth noting that the selection of an appropriate value of the *h* coefficient is important to make the phenomenon visible by simulation. Unfortunatelly, there is generally a lack of good material data in this area.

### 3.3. Geometry Effects

**Figure 21:**
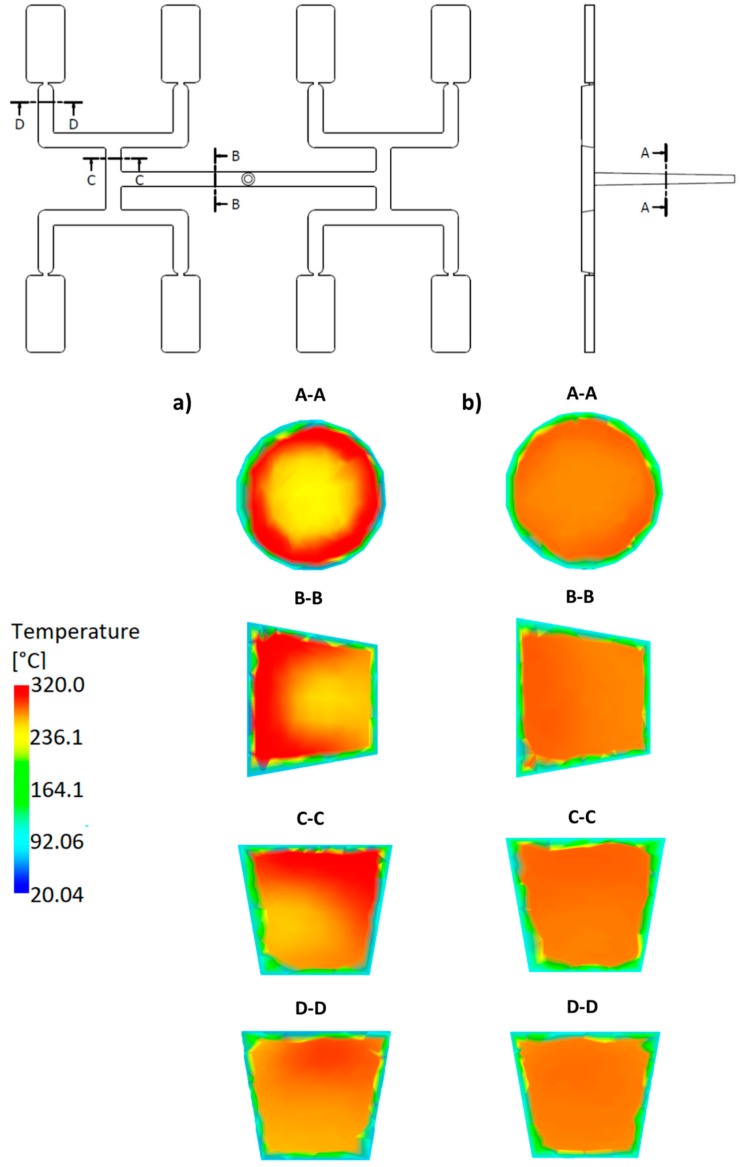
Temperature distribution in the selected cross-sections of the runners for different relaxation time at high injection rate in the case of the standard geometry *GS*: (**a**) λ = 0.001 s (*I_m_* = 20%), and (**b**) λ = 0.1 s (*I_m_* = 1%).

**Figure 22:**
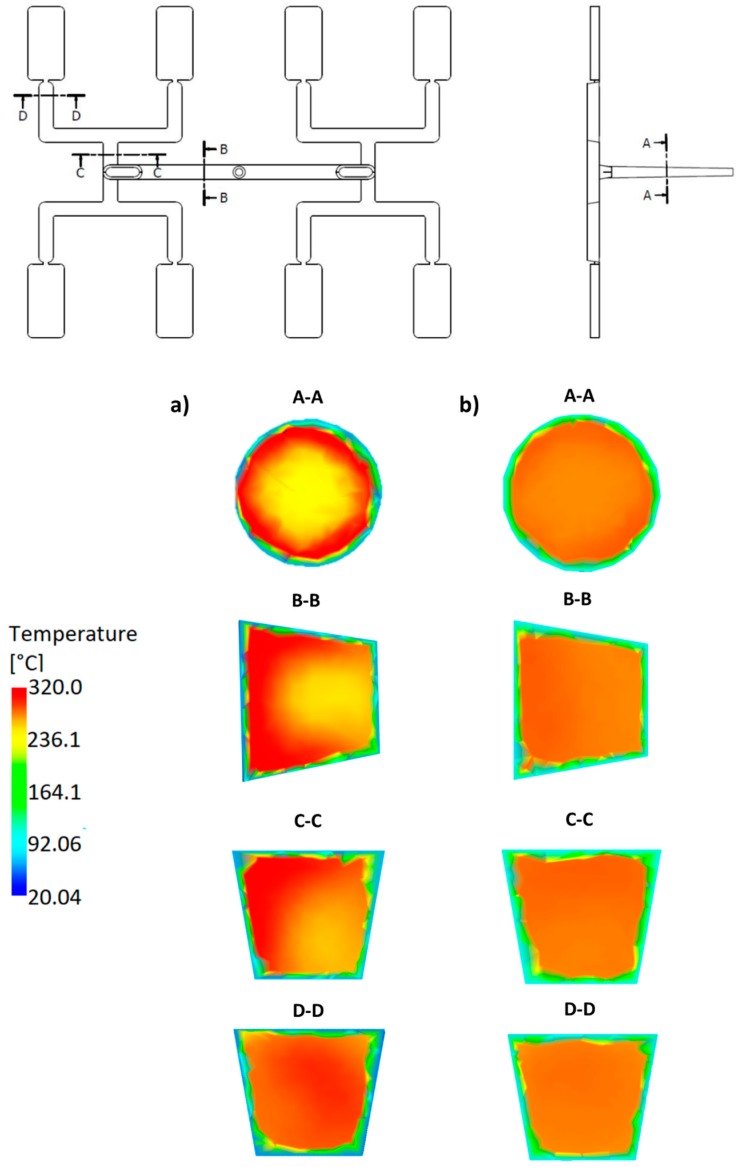
Temperature distribution in the selected cross-sections of the runners for different relaxation time at high injection rate in the case of the geometry with one overturn element *G1*: (**a**) λ = 0.001 s (*I_m_* = −35%), (**b**) λ = 0.1 s (*I_m_* = −1%).

**Figure 23:**
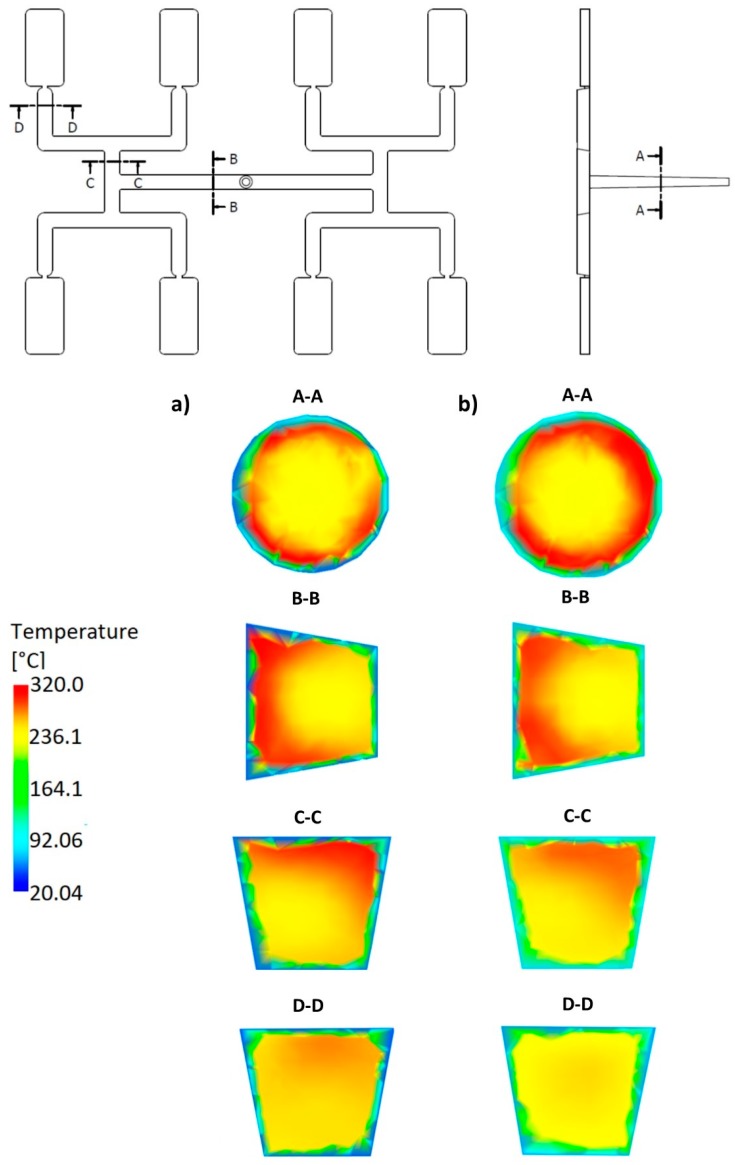
Temperature distribution in the selected cross-sections of the runners for different thermal diffusivity at high injection rate in the case of the standard geometry *GS*: (**a**) *α* = 0.005 mm^2^/s (*I_m_* = 22%), and (**b**) *α* = 5 mm^2^/s (*I_m_* = 12%).

**Figure 24:**
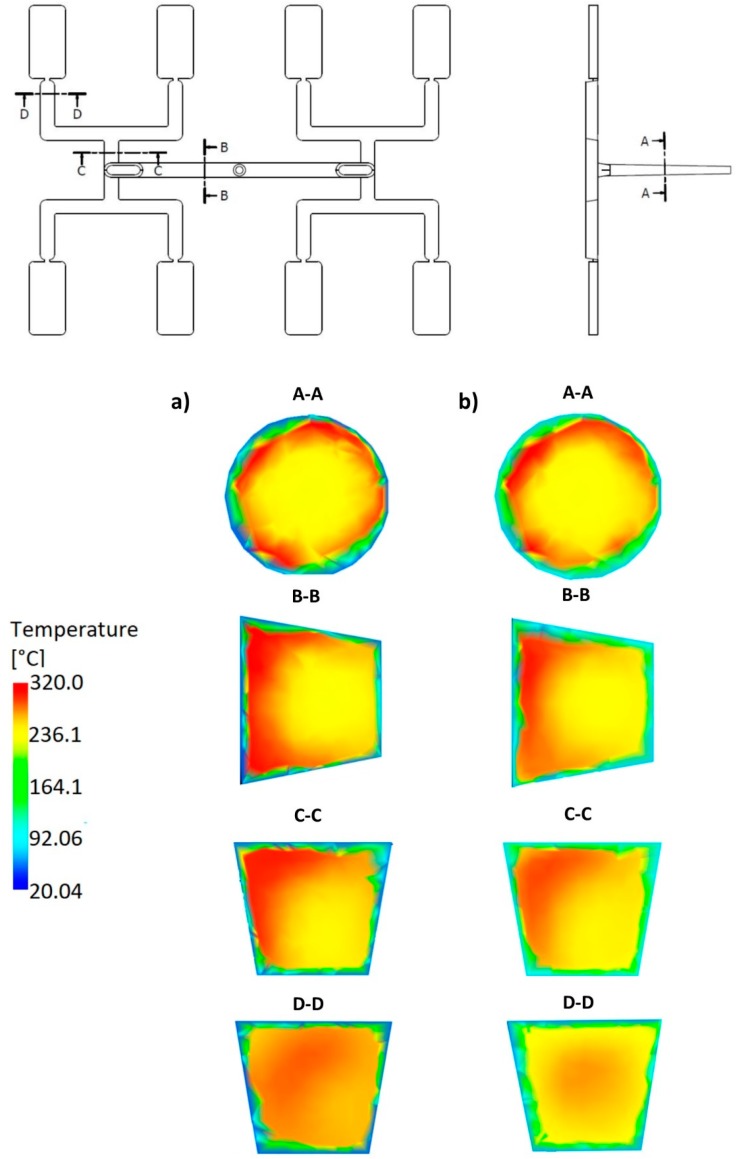
Temperature distribution in the selected cross-sections of the runners for different thermal diffusivity at high injection rate in the case of the geometry with one overturn element *G1*: (**a**) *α* = 0.005 mm^2^/s (*I_m_* = −33%), and (**b**) *α* = 5 mm^2^/s (*I_m_* = −17%).

An effect of the runner geometry for the selected rheological and thermal material parameters has been studied. The relaxation time λ and the thermal diffusivity α have been chosen for this. Here, as an example, the simulation results for the standard geometry *GS* of the runners layout, as well for the one overturn geometry *G1* have been presented for the high flow rate (500 cm^3^/s) (Figure 21, Figure 22, Figure 23 and Figure 24).

#### 3.3.1. The Relaxation Time

For the standard geometry *GS* (Figure 4a and Figure 21), the filling imbalance is positive (*I_m_* = 20%) for the low λ value (λ = 0.001 s) which means that inner cavities fills out faster, However, the imbalance decreases almost to zero (*I_m_* = 1%), when the relaxation time increases (λ = 0.1 s). This is consistent with Figure 13.

For the one overturn geometry *G1* (Figure 4b and Figure 22), the filling imbalance is negative (*I_m_* = −35%) for the low λ value (λ = 0.001 s) which means that outer cavities fills out faster, but the imbalance decreases basically to zero (*I_m_* = 1%), when the relaxation time increases (λ = 0.1 s).

Thus, in both cases, the geometry *GS* and *G1*, the imbalance is very high for the low relaxation time, and almost disappears for the high relaxation time. This results from a significant viscosity decrease with a relaxation time increase (Figure 6), i.e., the lower energy dissipation. This causes the flow with lower temperature gradients which does not promote the filling imbalance.

It is clearly seen that the high imbalance results from the high temperature gradients (Figure 21a and Figure 22a), and when the flow is balanced, the temperature gradients disappear (Figure 21b and Figure 22b).

It is also clearly seen that for the standard geometry *GS* the polymer melt stream rotates to the right (cross-section C-C, Figure 21a) which results in a positive imbalance. While, for the one overturn geometry *G1* the polymer melt stream rotates to the left (cross-section C-C, Figure 22a) which results in a negative imbalance. It is worth noting that for the high relaxation time (Figure 21a and Figure 22b) rotation is not observed.

#### 3.3.2. The Thermal Diffusivity

For the standard geometry *GS* (Figure 4a and Figure 23), the filling imbalance is positive (*I_m_* = 22%) for the low *α* value (*α* = 0.005 mm^2^/s) which means that inner cavities fills out faster. Additionally, it is also positive, but lower (*I_m_* = 12%), for the high *α* value (*α* = 5 mm^2^/s). This is consistent with Figure 17.

For the one overturn geometry *G1* (Figure 4b and Figure 24), the filling imbalance is negative (*I_m_* = −33%) for the low *α* value (*α* = 0.005 mm^2^/s) which means that outer cavities fills out faster. Additionally, it is also negative, but lower (*I_m_* = 12%), for the high *α* value (*α* = 5 mm^2^/s).

In both cases, the geometry *GS* and *G1*, the imbalance is higher for the low thermal diffusivity which results from the less intense heat exchange. The more intense heat exchange, i.e., the higher α promotes balancing the flow.

It is clearly seen that the high imbalance results from the high temperature gradients (Figure 23a and Figure 24a), and the imbalance decreases when lowering the temperature gradients (Figure 23b and Figure 24b).

It is also clearly seen that for the standard geometry *GS* the polymer melt stream rotates to the right in both cases, the low and high thermal diffusivity (cross-section C-C, Figure 23a,b) which results in a positive imbalance. While, for the one overturn geometry *G1* the polymer melt stream rotates to the left (cross-section C-C, Figure 24a,b) which results in a negative imbalance. It is worth noting that rotation is observed in all the cases, which clearly show that imbalance is connected with the temperature gradients and the melt stream rotation.

## 4. Conclusions

It has been observed that the Cross-WLF parameters, index flow, critical shear stress (relaxation time) and zero viscosity, as well as the thermal diffusivity and heat transfer coefficient strongly affect the filling imbalance. The effect is substantially dependent on the runners’ layout geometry as well as on the operating conditions, flow rate, and shear rate. Additionally, there are no simple rules for runners’ layout design. Generally, the standard runners system *GS* and the system with circled element *G3* induce a positive imbalance which means that inner cavities fills out faster, and it is opposite for the systems with one/two overturn elements *G1/G2* which cause negative imbalance. In general, for *GS* and *G3* systems, an effect of the zero shear rate viscosity η_0_ is positive (imbalance increases with an increase of viscosity), and an effect of the power law index *n* and the relaxation time λ is negative (imbalance decreases with an increase of index *n* and relaxation time λ). An effect of the thermal diffusivity α and heat transfer coefficient *h* is negative while an effect of the shear rate is positive. For *G1* and *G2* systems, the results of simulations indicate opposite dependencies. These observations have been summarized in Table 2.

It can be concluded, the velocity/shear rate distribution and the heat transfer conditions from the polymer melt to the mold are two main factors affecting the filling imbalance. It was observed that when flow rate increases, the filling imbalance increases since the non-linearity of velocity/shear rate distribution also increases. At low flow rates there is more time for the heat transfer from the polymer melt to the mold, and this may explain the imbalance increase with a decrease of the flow rate.

It can be summarized that filling imbalance problem is still unsolved. There are no universal runner layouts that could be successfully applied for mold design, and the design of runner systems is always dependent on the material characteristics and process parameters. If the design parameters of the runner system are inappropriate, the imbalance still happens. Hence, the assistance of simulation/optimization tools is desired to handle this phenomenon effectively.

The negative effects of mold filling imbalance are widely known in the industry, however, deterministic strategies of reducing this phenomenon are not available. This leads to the conclusion that only the statistical or AI (artificial intelligence) optimization techniques can improve the mold designing. We propose to use the STASA QC [55] system to optimize the filling imbalance applying the brain construction algorithm (BCA) to define a response surface of the problem. The melt injection rate, melt temperature, mold temperature, and the runner geometry will be optimized, and the global objective function will be defined by the filling imbalance coefficient (of the highest weight), injection pressure, molding temperature, and injection time. This concept will be presented in the next paper.

A comprehensive approach to modeling of the injection molding may be also useful for simulation of the flow in injection molds and for the prediction of filling imbalance. A global injection molding model might be considered for simulation of the polymer melt flow in the plasticating unit as well as in the mold. Resulting parameters of the plasticating unit simulations would be input data for the mold flow simulations.

## Figures and Tables

**Table 1:** Material Characteristics and Process Parameters.

**Injection molding process parameters**	
Mold temperature, *T*_mold_	63 °C
Coolant temperature, *T*_cool_	50 °C
Coolant Reynolds Number, *Re*	90,000
Melt temperature, *T*_melt_	252 °C
Anmbient temperature, *T*_amb_	20 °C
Flow rate, Q	5 cm^3^/s 500 cm^3^/s
Shear rate (at the wall, in the central runner), $\dot{\gamma }$	183.4 1/s 18,340 1/s
Injection/packing/cooling time, *t*_cycle_	10 s
Mold opening time, *t*_open_	5 s
**Material and rheological properties**	
Density	
- solid, ρ_s_	1204 kg/m^3^
- melt, ρ_m_	1016 kg/m^3^
MFI	13 g/10 min (5 kg, 250 °C)
Heat capacity	
- melt, *C*p_m_	2110 J/(kg °C)
- solid, *C*p_s_	1160 J/(kg °C)
Thermal conductivity - melt, *k*_m_	0.24 W/(m °C)
Melting temperature, *T*_m_	182 °C
Viscosity, η (Equation (1))	
Zero viscosity, η_0_ (Equation (2))	
- n	0.2139
- D_1_	5.32∙10^23^ Pa∙s
- τ*	353,100 Pa
- A_1_	59.833
- A_2_	51.6 K
- T^*^	323 K

**Table 2:** Simulation summary (green - *I_m_* *> 0*, red - *I_m_* *< 0*).

Effect of Process Parameters and Runners Layout on Filling Imbalance – Summary				
	*GS*	*G1*	*G2*	*G3*
*η_0_* ↗	↑	↓	↓	↑
*n* ↗	↓	↑	↑	↓
*λ* ↗	↓	↑	↑	↓
$\dot{\gamma }$ ↗	↑	↓	↓	↑
*α* ↗	↓	↑	↑	↓
*h* ↗	↓	↑	↑	↓
